# Diagnostic value of serum NLRP3, metalloproteinase‐9 and interferon‐γ for postoperative hydrocephalus and intracranial infection in patients with severe craniocerebral trauma

**DOI:** 10.1113/EP091463

**Published:** 2024-04-21

**Authors:** Qiang Peng, Lei Wang, Chun‐Mei Yu, Xin Chu, Bao‐Feng Zhu

**Affiliations:** ^1^ Department of Emergency Center The Second Affiliated Hospital of Nantong University (Nantong First People's Hospital) Nantong Jiangsu China

**Keywords:** diagnostic efficacy, hydrocephalus, independent risk factors, interferon‐γ, intracranial infection, metalloproteinase 9, NLRP3, severe craniocerebral trauma

## Abstract

Traumatic brain injury (TBI) is a major cause of morbidity and mortality globally. We unveiled the diagnostic value of serum NLRP3, metalloproteinase‐9 (MMP‐9) and interferon‐γ (IFN‐γ) levels in post‐craniotomy intracranial infections and hydrocephalus in patients with severe craniocerebral trauma to investigate the high risk factors for these in patients with TBI, and the serological factors predicting prognosis, which had a certain clinical predictive value. Study subjects underwent bone flap resection surgery and were categorized into the intracranial infection/hydrocephalus/control (without postoperative hydrocephalus or intracranial infection) groups, with their clinical data documented. Serum levels of NLRP3, MMP‐9 and IFN‐γ were determined using ELISA kits, with their diagnostic efficacy on intracranial infections and hydrocephalus evaluated by receiver operating characteristic curve analysis. The independent risk factors affecting postoperative intracranial infections and hydrocephalus were analysed by logistic multifactorial regression. The remission after postoperative symptomatic treatment was counted. The intracranial infection/control groups had significant differences in Glasgow Coma Scale (GCS) scores, opened injury, surgical time and cerebrospinal fluid leakage, whereas the hydrocephalus and control groups had marked differences in GCS scores, cerebrospinal fluid leakage and subdural effusion. Serum NLRP3, MMP‐9 and IFN‐γ levels were elevated in patients with post‐craniotomy intracranial infections/hydrocephalus. The area under the curve values of independent serum NLRP3, MMP‐9, IFN‐γ and their combination for diagnosing postoperative intracranial infection were 0.822, 0.722, 0.734 and 0.925, respectively, and for diagnosing hydrocephalus were 0.865, 0.828, 0.782 and 0.957, respectively. Serum NLRP3, MMP‐9 and IFN‐γ levels and serum NLRP3 and MMP‐9 levels were independent risk factors influencing postoperative intracranial infection and postoperative hydrocephalus, respectively. Patients with hydrocephalus had a high remission rate after postoperative symptomatic treatment. Serum NLRP3, MMP‐9 and IFN‐γ levels had high diagnostic efficacy in patients with postoperative intracranial infection and hydrocephalus, among which serum NLRP3 level played a major role.

## Abstract (highlights)

- **What is the central question of this study?**
  Severe craniocerebral injury usually leads to the loss of nerve function: what is the diagnostic value of serum NLRP3, metalloproteinase‐9 (MMP‐9) and interferon‐γ (IFN‐γ) levels in the diagnosis of intracranial infections and hydrocephalus in patients with severe craniocerebral trauma after craniotomy?
- **What is the main finding and its importance?**
  Serum NLRP3, MMP‐9, and IFN‐γ levels had high diagnostic efficacy in patients with postoperative intracranial infection and hydrocephalus, among which serum NLRP3 level played a major role.

## 1 INTRODUCTION

Traumatic brain injury (TBI) is defined as an acquired insult to the brain caused by an external mechanical force, which may contribute to temporary or permanent impairment (Capizzi et al., 2020). With the rapid development of industry and transportation, the rate of accidents is increasing year by year, accompanied by the increased number of patients with craniocerebral trauma, and severe traumatic brain injury (sTBI), as a common type of disease in neurosurgery, often results in the loss of nerve function, contributing to severe disturbance of consciousness, seizures, aphasia, hemiplegia and even a long‐term vegetative state (Li et al., 2022). Due to the rapid onset and development of craniocerebral trauma, patients need to be resuscitated in a timely manner, and craniotomy is one of the primary treatments for sTBI; it can effectively remove intracranial haematomas and reduce mortality, but is prone to postoperative hydrocephalus (Rufus et al., 2021; Wani et al., 2013). Hydrocephalus is a feature with excessive cerebrospinal fluid (CSF) accumulation in the brain ventricles, which often leads to cognitive and physical handicap (Krishnamurthy & Li, 2014). If left untreated, it will not only aggravate the craniocerebral injury, but also adversely impact the recovery of neurological function, and in severe cases, it can decrease brain hernia, jeopardizing the life of the patients. Besides, craniotomy can also cause intracranial infection (de Morais et al., 2021; Fang et al., 2017). Additionally, due to the long‐term exposure of brain tissues to air during craniotomy, bacteria and viruses can invade the patient's brain via the blood–brain barrier (BBB), which can easily initiate intracranial infection (de Morais et al., 2021; Fang et al., 2017). Intracranial infection stands out as one of the prevailing post‐sTBI complications, and also a critical risk factor for death (Yang et al., 2011). Therefore, prompt diagnosis of post‐craniotomy intracranial infection is essential to alleviate the prognosis of patients. Consequently, there is an urgent need to look for other multiple risk factors to diagnose hydrocephalus and intracranial infections, which will be of great significance for their reduction.

The determination of serum indicators has the advantages of accuracy, trace amount, short detection time and easy operation (Tang et al., 2021). As a pivotal factor in the innate immune response to tissue damage, the NLRP3 inflammasome is increasingly recognized as a driving factor for post‐haemorrhage injury (Heneka et al., 2018; Hoffman & Broderick, 2016). Notably, the NLRP3 inflammasomes possess the ability to release cytokines and aggregate brain oedema, neuronal apoptosis, as well as disruption of the blood–CSF barrier after intracranial haemorrhage (ICH) (Cheng et al., 2016; Groslambert & Py, 2018). Also, it has been demonstrated that suppression of microglia‐derived NLRP3 mitigates cognitive dysfunction and subependymal oedema in posthaemorrhagic hydrocephalus after ICH (Zhang, Guo et al., 2022). Furthermore, the activation of the immune inflammatory pathways (such as NLRP3) have been linked to brain injury, and thus the danger of intracerebral infection is elevated via overactivation of the inflammatory signalling (Jin et al., 2022). Matrix metalloproteinases (MMPs) are capable of degrading components of extracellular matrix (ECM), and macrophages are a critical source of MMPs (Zhang, Liu et al., 2019). Especially, MMP‐9 has been revealed to have an important role in pathophysiology, and either overexpression or dysregulation of MMP‐9 is related to various diseases (Mondal et al., 2020). MMP‐9 has previously been demonstrated to participate in the development and progression of TBI (Wei et al., 2021; Wu et al., 2020). Elevated MMP‐9 levels have been discovered in the CSF of haemorrhagic hydrocephalus children (Okamoto et al., 2010). Meanwhile, work has disclosed that MMP‐9 is elevated in the blood and CSF in sTBI patients (Grossetete et al., 2009). Another key focus of this study, interferon‐γ (IFN‐γ), as a crucial player in triggering cellular immunity, is able to orchestrate diverse protective functions to strengthen immune responses in cancers and infections (Kak et al., 2018). In the meantime, IFN‐γ may restrain angiogenesis in tumour tissues, trigger regulatory T cell apoptosis, and/or enhance the M1 proinflammatory macrophage activity to overcome tumour progression (Jorgovanovic et al., 2020). Moreover, a recent publication has unveiled that elevated IFN‐γ levels in the CSF of hydrocephalus patients may participate in advancing hydrocephalus progression (Lolansen et al., 2021). Besides, IFN‐γ has been discovered to be produced during intracranial infection (Lin et al., 2010). Regarding the expression levels and diagnostic value of serum NLRP3, MMP‐9 and IFN‐γ in patients with hydrocephalus and intracranial infections after craniotomy, no studies have been reported. Therefore, it is crucial to unravel expression levels and the diagnostic value of serum NLRP3, MMP‐9 and IFN‐γ in patients with hydrocephalus and intracranial infections after craniotomy, which will provide a reference for further clarifying the pathogenesis of hydrocephalus and intracranial infection, as well as new ideas for clinical prediction and management of hydrocephalus and intracranial infection.

## 2 METHODS

### 2.1 Ethical approval

All subjects were fully informed about the purpose of this study and signed an informed consent form before enrolment. The study conformed to the standards set by the *Declaration of Helsinki*, except for registration in a database.

### 2.2 Study subjects

One hundred and forty‐five patients with severe craniocerebral trauma admitted to The Second Affiliated Hospital of Nantong University (Nantong First People's Hospital) from January 2017 to June 2022 were recruited for the study, all of whom underwent bone flap resection surgery. According to whether hydrocephalus or intracranial infection occurred after surgery, the patients were categorized into the hydrocephalus group (*n* = 30), the intracranial infection group (*n* = 42) and the control group (*n* = 73). Patients in the control group had no postoperative complications of hydrocephalus or intracranial infection.

### 2.3 Inclusion and exclusion criteria

Inclusion criteria were as follows: (1) severe craniocerebral injury confirmed by head computed tomography (CT) or magnetic resonance imaging (MRI), with definite cause of trauma identified on admission; (2) Glasgow Coma Scale (GCS) score ≤8 points; (3) treated with bone flap resection; (4) no previous history of brain surgery; (5) showing hydrocephalus, defined as follows: hydrocephalus is an active distension of the ventricular system of the brain resulting from inadequate passage of cerebrospinal fluid from its point of production within the cerebral ventricles to its point of absorption into the systemic circulation (PMID: 18211712; PMID: 24932902), and high signal (MRI T_2_ image) or low density (CT) cerebrospinal fluid exudation around the enlarged periventricular space revealed by the brain MRI or CT diagnosis; (6) having intracranial infection defined as follows: clinical manifestations including headache, high fever, vomiting and positive result of CSF bacterial culture found by CSF puncture; and (7) complete clinical data.

Exclusion criteria were as below: (1) chronic intracranial haematoma; (2) severe complex injury and existing hydrocephalus; (3) signs of infection before surgery; (4) complication of malignant diseases; and (5) complication of severe organ dysfunction such as liver and kidney.

### 2.4 Data and sample collection

The following clinical baseline data were collected: age, sex, body mass index (BMI), cause of craniocerebral trauma (traffic accident, fall from height, blunt force trauma to the head, and external violent injury) (Zhang, Chen et al., 2019), GCS score at admission, site of injury (frontal lobe, temporal lobe and other sites), opened injury, time of surgery, postoperative CSF leakage, subdural effusion, and length of hospitalization. One day after surgery, 3 mL peripheral venous blood samples were collected, left for 30 min, and then centrifuged at 4°C and 845 *g* for 10 min. The supernatant was collected and divided into Eppendorf tubes, marked and kept at −80°C.

### 2.5 Enzyme linked immunosorbent assay

An enzyme linked immunosorbent assay (ELISA) kit (Milbio, Shanghai, China) was used to determine serum levels of NLRP3 (ml063259), MMP‐9 (ml058617m) and IFN‐γ (ml077386) in strict accordance with the kit requirements (Khaloo et al., 2020). Briefly, all the reagents were fully mixed, and the number of plates required was determined according to the number of samples to be tested plus the number of standard samples. The diluted standard samples and the samples to be tested were respectively added into the reaction wells, with the biotin‐labelled antibody immediately added. Then, the reaction well was covered with the membrane plate, and the samples were gently shaken and mixed well for subsequent incubation at 37°C. Ultimately, the ELISA plate was taken out, and the terminating solution quickly added, and the OD value of each well was determined at 450 nm. The sensitivity of NLRP3, MMP‐9 and IFN‐γ kits were as follows: the minimum detection concentrations were 1.0 pg/mL, 1.0 mg/L and 0.01 ng/mL, respectively; the specificity was as follows: all had no reaction with other cytokines; the repeatability was that the coefficients of variation of intra‐plate and inter‐plate were less than 10%.

### 2.6 Follow‐up

After 1 month and 12 months of the operation, 30 patients with severe craniocerebral injury after craniotomy complicated with hydrocephalus were reviewed and followed up. The disease remission after postoperative symptomatic treatment was recorded, and the total remission rate of symptoms was counted.

### 2.7 Statistical analysis

The data were analysed and plotted using SPSS 21.0 (IBM Corp. Armonk, NY, USA) and GraphPad Prism 8 (GraphPad Software, San Diego, CA, USA) statistical software. The Shapiro–Wilk (*W*) test was used to test whether the data were normally distributed, and data that conformed to normal distribution were expressed as means ± standard deviation. Comparisons between two groups were analysed by Student's unpaired *t*‐test, and comparisons along multiple groups were analysed by one‐way analysis of variance (ANOVA) with Tukey's test. Data that did not fit the normal distribution were expressed as median values (minimal value, maximal value) and tested using the Mann–Whitney test. Categorical variables were analysed using Fisher's exact test. The diagnostic value of serum NLRP3, MMP‐9 and IFN‐γ for intracranial infections and hydrocephalus was analysed using the receiver operating characteristic (ROC) curve. DeLong's test was utilized to analyse and compare the differences in the area under the curve (AUC). Logistic multifactorial regression analysis was implemented to assess the risk factors impacting intracranial infections and hydrocephalus. Differences were considered statistically significant at *P* < 0.05.

## 3 RESULTS

### 3.1 Comparisons of baseline data

A total of 145 study subjects were recruited in this study, all of whom underwent bone flap resection, and were categorized into the intracranial infection group (*n* = 42), the hydrocephalus group (*n* = 30), and the control group (*n* = 73) based on the presence or absence of hydrocephalus and intracranial infection in the postoperative period. Comparative analysis of the clinical baseline data of patients in the three groups revealed that there were no significant differences between the intracranial infection group and the control group in terms of age, sex, BMI, cause of craniocerebral trauma, site of injury, subdural effusion and length of hospitalization (all *P* > 0.05), whereas there were notable differences between the two groups in terms of GCS scores, opened injury, time of surgery and CSF leakage (all *P* < 0.05). No distinct differences were noted between the hydrocephalus group and the control group in terms of age, sex, BMI, cause of craniocerebral trauma, site of injury, opened injury, time of surgery and length of hospitalization (all *P* > 0.05), whereas there were significant differences when comparing them with respect to GCS scores, CSF leakage, and subdural effusion (all *P* < 0.05) (Table 1).

**TABLE 1:** Comparisons of general clinical data between patients of different severity and healthy subjects.

Characteristic	Control group (*n* = 73)	Intracranial infection group (*n* = 42)	Hydrocephalus group (*n* = 30)	*P*_a_	*P*_b_
Age (years)	46.36 ± 6.12	45.54 ± 6.03	44.54 ± 6.16	0.488	0.174
Sex (male, *n* (%))	44 (60.27)	24 (57.14)	17 (56.67)	0.844	0.826
BMI (kg/m^2^)	24.97 ± 2.64	25.06 ± 3.06	25.28 ± 3.14	0.869	0.610
Cause of craniocerebral trauma (*n* (%))					
Traffic accident	15 (20.54)	9 (21.43)	5 (16.67)	0.688	0.464
Fall from height	18 (24.66)	14 (33.33)	12 (40.00)		
Blunt force trauma	24 (32.88)	10 (23.81)	7 (23.33)		
External violent injury	16 (21.92)	9 (21.43)	6 (20.00)		
GCS score at admission	6 (4, 8)	5 (3, 6)	4 (2, 5)	< 0.0001	<0.0001
Site of injury (*n* (%))					
Frontal lobe	23 (31.51)	9 (21.43)	6 (20.00)	0.278	0.444
Temporal lobe	36 (49.32)	20 (47.62)	16 (53.33)		
Other sites	14 (19.17)	13 (30.95)	8 (26.67)		
Opened injury (*n* (%))					
Yes	21 (28.77)	20 (47.62)	14 (46.67)	0.0465	0.109
No	52 (71.23)	22 (52.38)	16 (53.33)		
Time of surgery (*n* (%))					
<4 h	41 (56.16)	14 (33.33)	13 (43.33)	0.0211	0.281
≥4 h	32 (43.84)	28 (66.67)	17 (56.67)		
Postoperative cerebrospinal fluid leakage (*n* (%))					
Yes	31 (42.47)	27 (64.29)	20 (66.67)	0.0330	0.0312
No	42 (57.53)	15 (35.71)	10 (33.33)		
Subdural effusion (*n* (%))					
Yes	24 (32.88)	16 (38.10)	17 (56.67)	0.685	0.0291
No	49 (67.12)	26 (61.90)	13 (43.33)		
Length of hospitalization (*n* (%))					
<10 d	39 (53.42)	19 (45.24)	12 (40.00)	0.442	0.279
≥10 d	34 (46.58)	23 (54.76)	18 (60.00)		

*Note*: Measurement data are expressed as means ± standard deviation or number of cases, and comparative analyses of categorical variables were performed using Fisher's exact test. Comparisons between two groups of continuous variables were analysed using Student's unpaired *t*‐test. GCS scores were expressed as median (minimal value, maximal value) values using the Mann–Whitney test. Differences were considered statistically significant at *P* < 0.05. *P*_a_: the intracranial infection group (*n* = 42) versus the control group (*n* = 73); *P*_b_: the hydrocephalus group (*n* = 30) versus the control group (*n* = 73).

Abbreviations: BMI, body mass index; GCS, Glasgow Coma Score.

### 3.2 Comparisons of postoperative serum NLRP 3, MMP‐9, and IFN‐γ levels in the three groups

**FIGURE 1:**
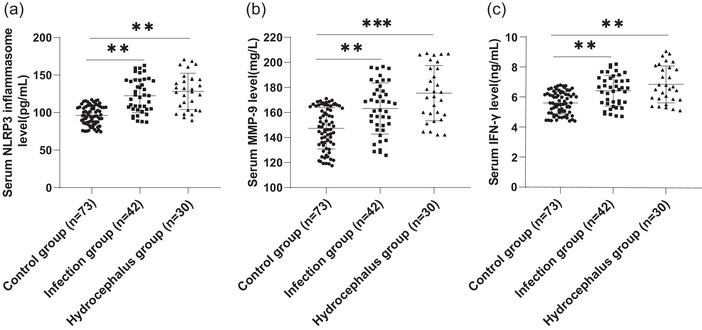
Comparisons of postoperative serum NLRP 3, MMP‐9 and IFN‐γ levels in the Control group (*n* = 73), the intracranial infection group (*n* = 42) and the hydrocephalus group (*n* = 30). (a–c) The levels of NLRP3, MMP‐9 and IFN‐γ in the serum of patients with craniocerebral trauma 1 day after craniotomy were examined by ELISA kits. The data are expressed as means ± standard deviation, and were compared among multiple groups using one‐way ANOVA with Tukey's test. **P < 0.01, ***P < 0.001.

It has been reported that the NLRP3 inflammasome, MMP‐9 and IFN‐γ promote the development and progression of hydrocephalus (Zhang, Tan et al., 2022). Therefore, we measured the levels of NLRP3, MMP‐9 and IFN‐γ in the serum of patients with craniocerebral trauma 1 day after craniotomy by ELISA kits, and found that the levels of NLRP3 inflammasome, MMP‐9, and IFN‐γ in the serum of patients with intracranial infection and hydrocephalus were significantly higher than those in the control group (all *P* < 0.01) (Figure 1a–c).

### 3.3 Serum NLRP3, MMP‐9 and IFN‐γ levels had high diagnostic efficacy in patients with postoperative intracranial infection and hydrocephalus

**FIGURE 2:**
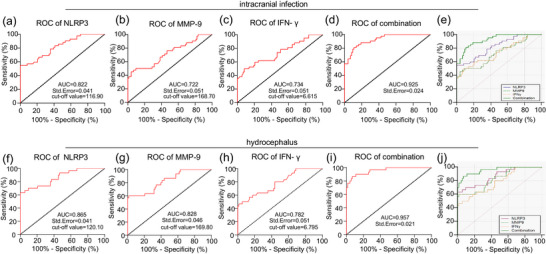
Serum NLRP3, MMP‐9 and IFN‐γ levels had high diagnostic efficacy in patients with postoperative intracranial infection and hydrocephalus. (a–d) Diagnostic efficacy of serum NLRP3, MMP‐9 and IFN‐γ levels and their combination in patients with postoperative intracranial infections was assessed separately by ROC curve analysis. (e) DeLong test of the differences in the AUC of NLRP3, MMP‐9 and IFN‐γ for the diagnosis of intracranial infection. (f–i) Diagnostic efficacy of serum NLRP3, MMP‐9 and IFN‐γ levels and their combination in patients with postoperative hydrocephalus was assessed separately by ROC curve analysis. (j) DeLong test of the differences in the AUC of NLRP 3, MMP‐9 and IFN‐γ for the diagnosis of hydrocephalus.

Subsequently, we evaluated the diagnostic efficacy of serum NLRP3, MMP‐9 and IFN‐γ levels for postoperative intracranial infection and hydrocephalus in patients by the ROC curve analysis. The results were that the AUC of serum NLRP3 levels for diagnosing intracranial infections in patients after surgery was 0.822, with a cutoff value of 116.90, a sensitivity of 54.76% and a specificity of 98.63%, demonstrating that serum NLRP3 levels >116.90 pg/mL could assist in the diagnosis of postoperative intracranial infections (Figure 2a). The AUC of serum MMP‐9 levels for diagnosing intracranial infections in patients after surgery was 0.722, with a cutoff value of 168.70, a sensitivity of 45.24% and a specificity of 95.89%, indicating that serum MMP‐9 levels >168.70 mg/L could assist in the diagnosis of po**s**toperative intracranial infections (Figure 2b). The AUC of serum IFN‐γ levels for diagnosing intracranial infections in patients after surgery was 0.734, with a cutoff value of 6.615, a sensitivity of 50.00% and a specificity of 91.78%, implying that serum IFN‐γ levels >6.615 ng/mL could assist in the diagnosis of postoperative intracranial infections (Figure 2c). In addition, we observed that the AUC for the combined diagnosis of intracranial infections by serum NLRP3, MMP‐9 and IFN‐γ was 0.925 (Figure 2d), and DeLong's test of the differences in the AUC showed that the diagnostic efficacy of the combination of the three for intracranial infections was higher than the diagnostic efficacy of NLRP3, MMP‐9 or IFN‐γ alone (Figure 2e). Additionally, the model is reported to have a certain degree of robustness when AUC is 0.75, but have slightly lower performance when AUC is <0.75 (Meyer et al., 2018). As a result, we speculated that serum NLRP3 level might play a principal driving role in the AUC value of the combined diagnosis of the three for intracranial infection.

In the meantime, the AUC of serum NLRP3 levels for diagnosing the occurrence of postoperative hydrocephalus in patients was 0.865 (120.10 cutoff value, 63.33% sensitivity, and 100.00% specificity), suggesting that serum NLRP3 level >120.10 pg/mL could assist in the diagnosis of postoperative hydrocephalus (Figure 2f), that of serum MMP‐9 levels was 0.828 (169.80 cutoff value, 60.00% sensitivity and 98.63% specificity), revealing that serum MMP‐9 level >169.80 mg/L could assist in the diagnosis of postoperative hydrocephalus (Figure 2g), and that of serum IFN‐γ levels was 0.782 (cutoff value 6.795, sensitivity 46.67% and specificity 98.63%), indicating that serum IFN‐γ level >6.795 ng/mL could be an aid in the diagnosis of postoperative hydrocephalus (Figure 2h). Similarly, the AUC of the combination of serum NLRP3, MMP‐9 and IFN‐γ for the diagnosis of hydrocephalus was 0.957 (Figure 2i), and the diagnostic efficacy of the combination of the three for hydrocephalus was higher than the diagnostic efficacy of NLRP3, MMP‐9 or IFN‐γ alone (Figure 2j). Overall, serum NLRP3, MMP‐9 and IFN‐γ levels had high diagnostic efficacy for postoperative intracranial infection and hydrocephalus in patients.

### 3.4 Logistic multifactorial regression analysis of independent risk factors affecting postoperative intracranial infection and hydrocephalus in patients with craniocerebral trauma

Hydrocephalus is a common complication after craniocerebral injury, and it is also the main cause of headache, mental retardation and dementia in patients after surgery. Exploring the risk factors influencing the occurrence of intracranial infections and hydrocephalus in patients with craniocerebral injuries after surgery can effectively reduce the incidence of intracranial infections and hydrocephalus in patients after surgery (Wirth & Sobey, 2006). First, we included whether the patients developed intracranial infections after surgery as the dependent variable, and combining with the results of the analysis in Table 1 (*P* < 0.05 for the independent variables), included the GCS score at admission, opened injury, time of surgery, postoperative CSF leakage, and serum levels of NLRP3, MMP‐9 and IFN‐γ as the independent variables in a logistic multifactorial regression analysis model. The findings disclosed that GCS score at admission (*P* = 0.003, odds ratiio (OR) = 0.387, 95% CI: 0.205–0.730), serum NLRP3 level (*P* = 0.001, OR = 1.099, 95% CI: 1.041–1.161), serum MMP‐9 level (*P* = 0.006, OR = 1.068, 95% CI:1.019–1.119) and serum IFN‐γ level (*P* = 0.010, OR = 3.754, 95% CI: 1.368–10.300) were independent risk factors impacting postoperative intracranial infections in patients (Table 2).

**TABLE 2:** Logistic multifactorial regression analysis of independent risk factors influencing the postoperative intracranial infections in patients (*n* = 42).

Variable	*P*	OR (95%CI)
GCS score at admission	0.003	0.387 (0.205–0.730)
Opened injury	0.522	1.694 (0.338–8.488)
Time of surgery	0.174	2.868 (0.627–13.118)
Postoperative cerebrospinal fluid leakage	0.055	4.869 (0.969–24.476)
NLRP3 (pg/mL)	0.001	1.099 (1.041–1.161)
MMP‐9 (mg/L)	0.006	1.068 (1.019–1.119)
IFN‐γ (ng/mL)	0.010	3.754 (1.368–10.300)

*Note*: Differences were considered statistically significant at *P* < 0.05. Abbreviations: CI, confidence interval; GCS, Glasgow coma score; IFN‐γ, interferon‐γ; MMP‐9, matrix metallopeptidase‐9; NLRP3, nucleotide‐binding oligomerization domain‐like receptor protein 3; OR, odds ratio.

For hydrocephalus, the GCS score at admission, postoperative CSF leakage, subdural effusion, and serum NLRP3, MMP‐9 and IFN‐γ levels were included in the logistic multifactorial regression analysis model, with whether the patients developed hydrocephalus after surgery as the dependent variable, and combining the results of the analyses in Table 1 (*P *< 0.05 for the independent variables). It was demonstrated that GCS score at admission (*P* = 0.028, OR = 0.082, 95% CI: 0.009–0.765), serum NLRP3 level (*P* = 0.044, OR = 1.160, 95% CI: 1.004–1.341) and serum MMP‐9 level (*P* = 0.011, OR = 1.143, 95% CI: 1.030–1.268) were all independent risk factors affecting the patients’ postoperative hydrocephalus (Table 3).

**TABLE 3:** Logistic multifactorial regression analysis of independent risk factors influencing the postoperative hydrocephalus in patients (*n* = 30).

Variable	*P*	OR (95%CI)
GCS score at admission	0.028	0.082 (0.009–0.765)
Postoperative cerebrospinal fluid leakage	0.155	0.055 (0.001–2.987)
Subdural effusion	0.220	5.251 (0.370–74.525)
NLRP3 (pg/mL)	0.044	1.160 (1.004–1.341)
MMP‐9 (mg/L)	0.011	1.143 (1.030–1.268)
IFN‐γ (ng/mL)	0.081	6.872 (0.789–59.835)

*Note*: Differences were considered statistically significant at *P* < 0.05. Abbreviations: CI, confidence interval; GCS, Glasgow coma score; IFN‐γ, interferon‐γ; MMP‐9, matrix metallopeptidase‐9; NLRP3, nucleotide‐binding oligomerization domain‐like receptor protein 3; OR, odds ratio.

### 3.5 Remission after postoperative symptomatic treatment in patients with hydrocephalus

Follow‐up of 30 patients with hydrocephalus after craniotomy for severe craniocerebral injuries at 1 month revealed that the ventricles of the brain were reduced to different degrees in nine patients, the ventricles of the brain were restored to their normal shape in 12 patients, remission was complete (light, moderate‐to‐severe) in three patients, and remission did not happen in six patients, with a total remission rate of 80.00% (24/30). When the patients were followed up after 12 months, 10 patients had different degrees of reduction of the cerebral ventricles, 13 patients had the ventricles returned to normal, two patients with light hydrocephalus had complete remission of their symptoms, three patients with moderate‐to‐severe hydrocephalus had complete remission of their symptoms, and two patients had no remission of their symptoms; there were no vegetative or fatal cases, and the overall symptom remission rate was 93.33% (28/30) (Table 4). These results indicated that patients with hydrocephalus had a high rate of remission after postoperative symptomatic treatment.

**TABLE 4:** Remission after postoperative symptomatic treatment in patients with hydrocephalus (*n* = 30).

Remission status	One month after surgery	12 month after surgery
Different degrees of reduction of the cerebral ventricles (effective)	9	10
The ventricle returned to normal (markedly effective)	12	13
Complete remission (light)	1	2
Complete remission (moderate and severe)	2	3
Insignificant remission (moderate and severe) (no remission)	6	2
Person in coma or death (ineffective)	0	0
Total (%)	24 (80.00%)	28 (93.33%)

## 4 DISCUSSION

Severe craniocerebral injury contributes to elevated secretion of catecholamines, glucagon and glucocorticoid, reduced insulin secretion, and advanced body catabolism and energy consumption (Wang et al., 2021). Patients with craniocerebral injury often have varying degrees of cerebral oedema, and intracranial pressure elevation resulting from severe cerebral oedema is a fatal factor that needs much attention (Kostic et al., 2011). Craniotomy is a primary therapy for severe craniocerebral injury, but is prone to cause postoperative hydrocephalus and intracranial infection (Fang et al., 2017; Rufus et al., 2021). Intracranial infection is a frequent postoperative complication in neurosurgical patients, and if not treated quickly, it contributes to substantial morbidity and mortality (Yang et al., 2011). Besides, hydrocephalus can also lead to poor outcome and clinical deterioration if untreated (Kammersgaard et al., 2013). Herein, we aimed to unveil the diagnostic value of serum NLRP3, MMP‐9 and IFN‐γ levels in the diagnosis of intracranial infections and hydrocephalus in patients with severe craniocerebral trauma after craniotomy, and our findings highlighted that serum MMP‐9, NLRP3 and IFN‐γ levels were linked to the risk of hydrocephalus and intracranial infections after craniotomy in patients with severe craniocerebral trauma.

In our research, it was demonstrated that serum NLRP3 levels were significantly elevated in intracranial infections and hydrocephalus in post‐craniotomy patients. Besides, NLRP3 had a high diagnostic efficacy for postoperative intracranial infections and hydrocephalus in patients, and it was also an independent risk factor influencing patients’ postoperative intracranial infection and hydrocephalus. The NLRP3 inflammasomes boost the inflammatory response by advancing neutrophil infiltration and releasing IL‐1β following ICH (Ma et al., 2014). Recent evidence has disclosed that the ICH‐evoked activation of the NLRP3 inflammasome can strengthen the inflammatory response, thereby aggravating brain oedema (Ren et al., 2018). Yao et al. (2017) have also declared that complement‐triggered ICH neuroinflammation depends on the activation of NLRP3, which promotes lipopolysaccharide‐ and ICH‐induced neuroinflammation, and NLRP3 is necessary for ICH‐induced inflammation. Besides, another study has demonstrated that NLRP3 activation in the choroid plexus leads to elevated CSF secretion and depraved hydrocephalus after ICH with ventricular extension (Zhang, Tan et al., 2022).

Several MMPs, including MMP‐9, are implicated in neuroregeneration, neurodevelopment and some pathological conditions (Yong et al., 2001). In light of this, our findings revealed that serum MMP‐9 levels were significantly elevated in post‐craniotomy patients with hydrocephalus and intracranial infections. Besides, MMP‐9 had a high diagnostic efficacy for postoperative intracranial infections and hydrocephalus in patients, which was also an independent risk factor influencing patients’ postoperative hydrocephalus and intracranial infection. Likewise, increased levels of MMPs have been found in TBI and may result in additional tissue injury together with BBB damage (Minta et al., 2020). Ventricular dilatation after intraventricular haemorrhage (IVH) has been revealed to result from the widespread deposition of ECM proteins in the subarachnoid space, which can be degraded by MMP‐9, implying that MMP‐9 possibly functions in the resolution of progressive ventricular dilation post‐IVH (Okamoto et al., 2010). Similar to our findings, MMP‐9 expression in the cerebral tissues and CSF is increased in hydrocephalus, indicating that MMP‐9 may be implicated in hydrocephalus after kaolin induction (Zhang et al., 2013).

The levels of inflammatory cytokines in CSF and serum are elevated in patients with hydrocephalus, hydrocephalus patients exhibit signs of inflammation, and IFN‐γ intensifies the inflammatory response once inflammation is initiated (Sosvorova et al., 2015; UlFigur, 2004). As for serum IFN‐γ, our findings disclosed its levels were markedly elevated in patients with intracranial infections and hydrocephalus after craniotomy as well. What is more, IFN‐γ had good diagnostic efficacy for postoperative hydrocephalus and intracranial infections in patients, and was also an independent risk factor influencing patients’ postoperative intracranial infection. Consistently, the increased IFN‐γ levels in patients with hydrocephalus diagnoses demonstrate an ongoing inflammatory condition (Lolansen *et al.*, 2021). Higher IFN‐γ levels are discovered in patients developing cystic white matter damage, signifying its participation in cystic white matter damage pathogenesis in the context of posthaemorrhagic hydrocephalus (Schmitz *et al.*, 2007). Also, Lolansen et al. (2021) have found that IFN‐γ is elevated in CSF of hydrocephalus patients, which may serve as a new disease biomarker for hydrocephalus.

Taken together, this work underlines that the levels of NLRP3, IFN‐γ and MMP‐9 are markedly increased in the serum of patients with intracranial infections and hydrocephalus after craniotomy, and their combined diagnostic efficacy is the highest for postoperative intracranial infections and hydrocephalus in patients. This paper provides a new reference for the diagnosis of intracranial infections and hydrocephalus after craniotomy. Nevertheless, larger multicentre studies with expanded sample size and matched controls are warranted to further clarify the diagnostic capacity of NLRP3, IFN‐γ and MMP‐9. Moreover, the molecular regulatory mechanism of them and other cytogenic factors in the development of hydrocephalus needs to be further elucidated.

## AUTHOR CONTRIBUTIONS

All authors contributed to the study conception and design. Qiang Peng and Bao‐Feng Zhu contributed to the guarantor of integrity of the entire study and manuscript review; Qiang Peng and Lei Wang contributed to the study concepts, study design, and manuscript preparation; Chun‐Mei Yu, and Xin Chu contributed to the definition of intellectual content and literature research; Qiang Peng, Chun‐Mei Yu, and Bao‐Feng Zhu contributed to the clinical studies and experimental studies; Chun‐Mei Yu and Xin Chu contributed to the data acquisition; Qiang Peng and Lei Wang contributed to the data analysis; Qiang Peng, Chun‐Mei Yu, and Xin Chu contributed to the statistical analysis. All authors have read and approved the final version of this manuscript and agree to be accountable for all aspects of the work in ensuring that questions related to the accuracy or integrity of any part of the work are appropriately investigated and resolved. All persons designated as authors qualify for authorship, and all those who qualify for authorship are listed.

## CONFLICT OF INTEREST

The authors have no relevant financial or non‐financial interests to disclose.

## Data Availability

The data that support the findings of this study are available from the corresponding author upon reasonable request.
